# Diagnostic and therapeutic delay in patients with larynx cancer at a reference public hospital

**DOI:** 10.1590/S1808-86942010000600005

**Published:** 2015-10-19

**Authors:** Ali Amar, Helma Maria Chedid, Sergio Altino Franzi, Abrão Rapoport

**Affiliations:** 1 PhD - UNIFESP, Head and Neck Surgeon - Hospital Heliópolis; 2 MSc - HOSPHEL, Head and Neck Surgeon - Hospital Heliópolis; 3 PhD - USP, Head and Neck Surgeon - Hospital Heliópolis; 4 Associate Professor - USP, Head and Neck Surgeon - Hospital Heliópolis. Hospital Heliópolis

**Keywords:** carcinoma, squamous cell, laryngeal neoplasms

## Abstract

Laryngeal squamous cell carcinoma is very often diagnosed at advanced stages. The time interval between the specialist consultation and the start of treatment may contribute to better outcomes.

**Aim:**

the interval assessment between the first specialist evaluation and the treatment of patients with laryngeal squamous cell carcinoma.

**Study design:**

longitudinal historical cohort.

**Materials and Methods:**

272 consecutive patients with laryngeal squamous cell carcinoma seen between January, 1996 and December of 2004. Clinical and epidemiological data were evaluated, as well as their association with the time interval between the first specialist visit and the start of treatment.

**Result:**

the median time between first evaluation and treatment was 49 days. There was no relationship with gender, age, birth place, disease stage or education.

**Conclusion:**

the treatment median delay was 49 days, similar to what has been reported in other studies.

## INTRODUCTION

Laryngeal cancer represents 2% of the cancer cases in Brazil, with estimates of 9,320 new cases in the year of 2009, according to the National Cancer Institute -INCA^1^.

Epidermoid carcinoma is the most common histological type and, despite the early symptoms, it is still frequently diagnosed in an advanced stage. Diagnosis and treatment delays can be broken down in two stages. The first stage, until it reaches specialized care, was influenced both by the patient who denied symptoms as well as delay in primary care. Another stage is diagnosis and treatment themselves. Although the time spent in the first stage is usually longer, being responsible for the advanced stage of the disease, delays in starting treatment may also bring about a worse outcome^2^, ^3^. The present investigation aims at checking the time taken after the patient sees the specialist all the way to treatment onset, as well as its relationship with some clinical and epidemiological characteristics of the patients seen in a reference public medical care facility.

## MATERIALS AND METHODS

This study is a series of retrospective cases, involving the review of the medical charts from 272 patients with laryngeal epidermoid carcinoma, consecutively seen between January of 1996 and December of 2004. We assessed the time interval between the first patient visit to the head and neck surgery department and surgical treatment or radiotherapy onset, as well as its association with clinical and epidemiological characteristics, including staging, gender, age, education and place of birth. Moreover, we also assessed treatment delays at different times (three years). The continuous quantitative variable (time) did not have a normal distribution, confirmed by the Kolmogorof-Smirnoff test; thus, we employed the Mann-Whitney and Kruskal-Wallis non-parametric tests, considering significant those differences with p<0.05. The time intervals were expressed in medians, quartiles and percentiles. This study was approved by the Ethics in Research Committee of the institution - record # 659.

## RESULTS

Patients averaged 58 years of age (36 to 83 years), 237 males and 35 females. The complaint time averaged 6 months. Forty-seven percent had been born in the state of São Paulo, 70% were illiterate or had not even finished junior high school; and 63% had advanced primary tumor (T3 or T4). As far as treatment is concerned, 132 were submitted to surgery and 85 to radiotherapy. Fifty-five patients were not followed up, and 19 were not treated either with surgery or radiotherapy because of not being clinically fit, they refused to do it or died. Twenty-seven patients did not return for treatment, six others were referred to radiotherapy and did not return for scheduled follow up and three patients chose to undergo treatment in another institution.

**Figure 1:**
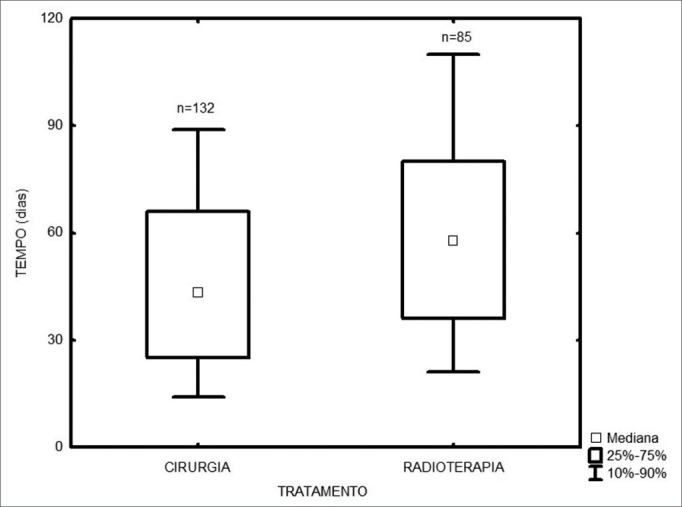
Time interval between the first medical visit and treatment onset according to treatment mode.

**Figure 2:**
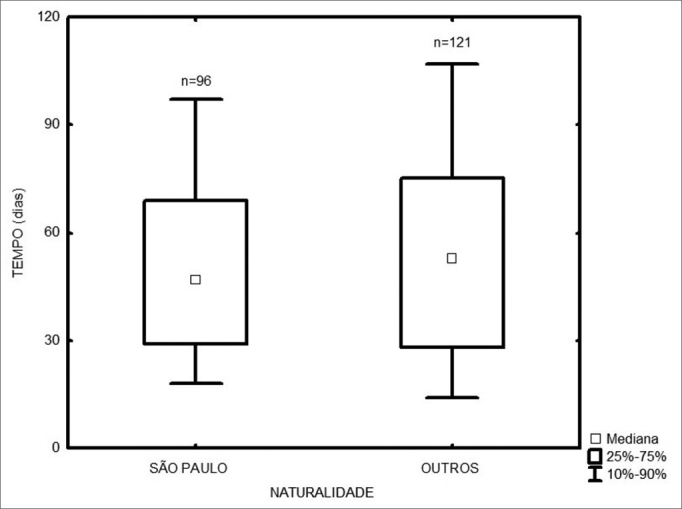
Time interval between the first medical visit and treatment onset according to patient place of birth.

**Figure 3:**
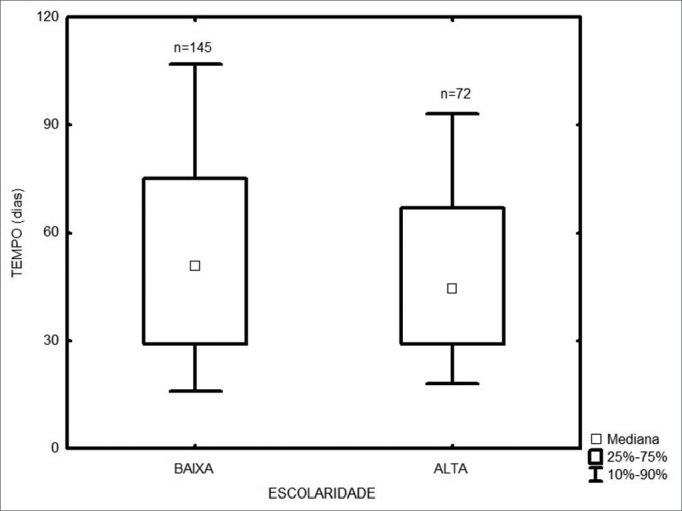
Time interval between the first medical visit and treatment onset according to patient educational level.

**Figure 4:**
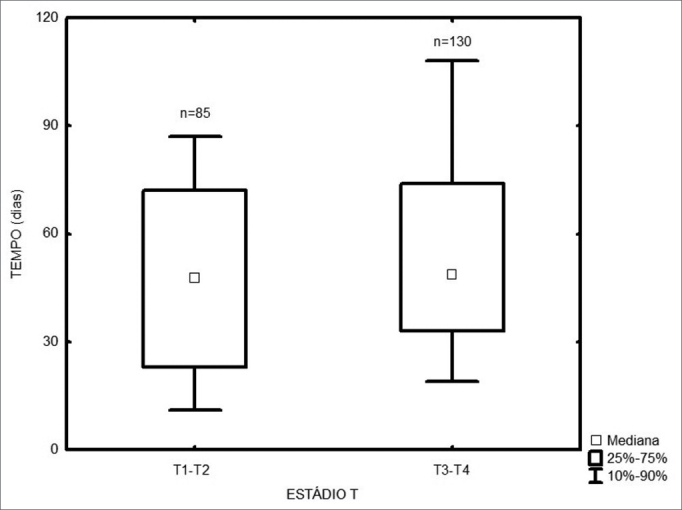
Time interval between the first medical visit and treatment onset according to the T stage.

**Figure 5:**
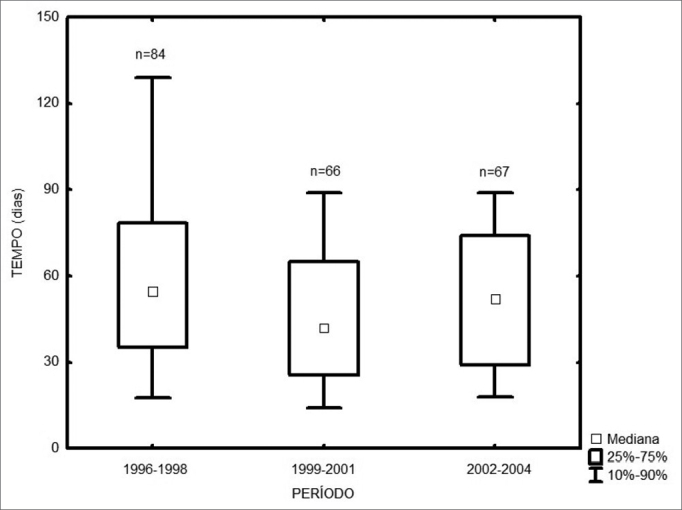
Time interval between the first medical visit and treatment onset in the different three-year periods.

**Figure 6:**
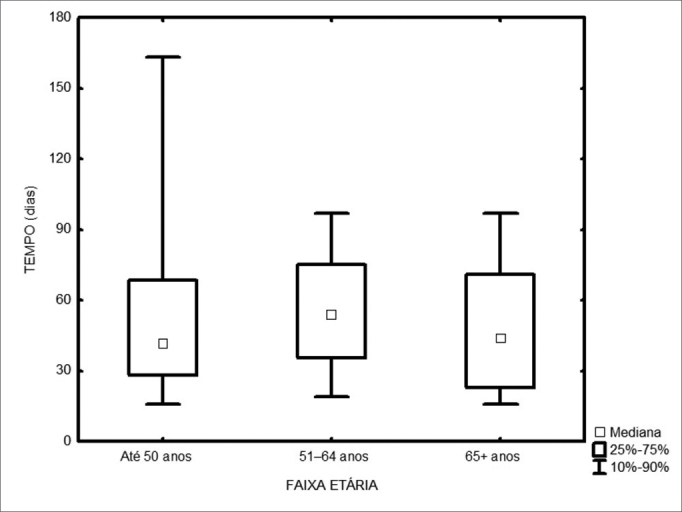
Time interval between the first medical visit and treatment onset according to age.

Among the 217 treated patients, the time interval between the first medical visit and treatment onset varied from 1 to 347 days, with a median of 49 days. The radiotherapy onset median time was 58 days, and for surgery it was 43 days (Figure 1). As far as place of birth is concerned, those patients from São Paulo took 47 days to start treatment, and those from other states took 53 days (Figure 2, p=0.49). Illiterate patients or those who did not finish junior high school or with more school years than that took 51 days, while those who finished high school or higher education took a median of 46 days (Figure 3, p=0.33). As far as staging is concerned, patients with T1 or T2 tumors took 47 days, those with T3 or T4 tumors took a median of 49 days to start treatment (Figure 4, p=0.28). In the three different three-year intervals analyzed, between 1996 and 1998 the median was of 54 days; between 1999 and 2001 it was of 42 days and between 2002 and 2004 it was of 52 days (Figure 5, p=0.09). The male patients had median waiting time of 48 days and the females had 53 days (p=0.29). As far as age is concerned, we also did not find differences in treatment waiting time (Figure 6, p=0.24).

## DISCUSSION

The diagnosis of laryngeal malignant neoplasia is based on pathology tests. Since the biopsy of these lesions requires endoscopy or direct laryngoscopy, the ultimate diagnosis is usually done by the consultant. During the time period evaluated, almost all the biopsies were done through direct laryngoscopy under general anesthesia, which may have increased the pretreatment time. As far as radiotherapy goes, which we did not have available, the time to treatment also counts in the referral to another clinic, however with the patient properly staged and with histological diagnosis. This study did not aim at comparing the time taken between the two treatment options, and rather to record the time-to-treatment interval for patients who came to a specialized clinic. According to Primdahl et al., currently more image studies are ordered and radiotherapy has a more complex planning, however they mention equipment availability (linear accelerator) as a preponderant factor in the greater delay seen upon treatment onset, which in Denmark increased from 50 to 70 days between 1992 and 2002^2^. These authors estimated that the increase of 20 days could reduce control rates in 10%^2^. Brouha et al. reported a median interval of 49 days between the first consultation in one specialized clinic in Holland and treatment onset, and such interval was greater in laryngeal tumors^4^. Undoubtedly, treatment waiting depends on the availability of human and material 703 resources - depending on the infrastructure of the surgical center or radiotherapy. In a general hospital, resources are usually limited, treatment priorities may also cause conflicts. In a system working at full capacity, mild seasonal variations in disease incidence or temporary equipment unavailability may intensely reflect on this time interval. Despite the growing service complexity, which today embodies multidisciplinary teams and standardizes a greater number of complementary methods, many stages can be ran simultaneously, which enables patient preparation in a shorter time interval. Considering the constant pursue of treatment gains, one must appreciate the possibility of shortening treatment waiting time. This is a doable goal, which can be reached with the rational use of resources. Unfortunately, the delay in the pre-diagnostic phase cannot be tackled by the medical team. Although the time of symptoms is usually longer than the delay in diagnosis and treatment, according to Allison et al., a delay of more than one month in primary care is associated with the advanced-stage disease, and we did not notice an association with the time of complaint^4^. Teppo et al. associate a diagnostic delay and not the time of complaint as specific determining factor on the specific-disease survival^5^. Other authors also suggest that the treatment delay may influence the prognosis of these patients, either directly or indirectly. Epidermoid carcinomas may have different tumor duplication times, but they invariably grow, and tumor enlargement has direct impact on tumor surgical or radiotherapy treatment. Tumor growth may require greater resection in a noble area, with its consequences in reconstruction and morbidity, it may even become impossible to resect. Equally important, the radiotherapy control rate is associated with tumor volume. Kowalski and Carvalho noticed that the patients with increments in TNM staging before treatment had the worst result, when paired with patients who kept the initial staging^6^. Barton et al., evaluated initial laryngeal tumors and did not observe pre-treatment time influence on prognosis; nonetheless, the waiting time assessed between the biopsy and radiotherapy onset had a median time of only 24 days^7^. On the other hand, Jensen et al. reported a median increase of 46% in tumor volume in 62% of the patients awaiting treatment onset after the 28 day median^8^. This latest study may have overestimated tumor growth by means of patient-selection, since only patients with image exams repeated at different dates prior to treatment were included.

Most patients had low education levels and advanced disease. The results show that there was no treatment priority based on clinical or epidemiological characteristics. Surprisingly, treatment delay was similar to what had been reported by developed countries^2^, ^4^. Approximately 10% of the patients did not return after the first visit, and because of that we do not know whether they refused treatment or if they were seen in another institution. The 49-day delay (median) for treatment onset is exaggerated, since half of the patients were treated after a time interval equal to or greater than this. Considering the possible tumor growth in this period, to reduce such time may impact prognosis similarly or even higher than that of the use of new technologies.

## CONCLUSION

The time between initial care with the specialist and treatment onset had a median value of 49 days, similar to what has been reported in other studies, but higher that what is adequate for cancer treatment.
